# Effect of a Mobile Health–Based Remote Interaction Management Intervention on the Quality of Life and Self-Management Behavior of Patients With Low Anterior Resection Syndrome: Randomized Controlled Trial

**DOI:** 10.2196/53909

**Published:** 2024-08-13

**Authors:** Peng Zhou, Hui Li, Xueying Pang, Ting Wang, Yan Wang, Hongye He, Dongmei Zhuang, Furong Zhu, Rui Zhu, Shaohua Hu

**Affiliations:** 1 Department of Nursing the First Affiliated Hospital of Anhui Medical University Hefei China; 2 School of Nursing Anhui Medical University Hefei China; 3 College of Traditional Chinese Medicine Bozhou University Bozhou China; 4 Department of Gastrointestinal Surgery the First Affiliated Hospital of Anhui Medical University Hefei China

**Keywords:** mHealth, low anterior resection syndrome, quality of life, sphincter-preserving surgery, randomized controlled trial, mobile health, mobile phone

## Abstract

**Background:**

People who undergo sphincter-preserving surgery have high rates of anorectal functional disturbances, known as low anterior resection syndrome (LARS). LARS negatively affects patients’ quality of life (QoL) and increases their need for self-management behaviors. Therefore, approaches to enhance self-management behavior and QoL are vital.

**Objective:**

This study aims to assess the effectiveness of a remote digital management intervention designed to enhance the QoL and self-management behavior of patients with LARS.

**Methods:**

From July 2022 to May 2023, we conducted a single-blinded randomized controlled trial and recruited 120 patients with LARS in a tertiary hospital in Hefei, China. All patients were randomly assigned to the intervention group (using the “e-bowel safety” applet and monthly motivational interviewing) or the control group (usual care and an information booklet). Our team provided a 3-month intervention and followed up with all patients for an additional 3 months. The primary outcome was patient QoL measured using the European Organization for Research and Treatment of Cancer Quality of Life Questionnaire Core 30. The secondary outcomes were evaluated using the Bowel Symptoms Self-Management Behaviors Questionnaire, LARS score, and Perceived Social Support Scale. Data collection occurred at study enrollment, the end of the 3-month intervention, and the 3-month follow-up. Generalized estimating equations were used to analyze changes in all outcome variables.

**Results:**

In the end, 111 patients completed the study. In the intervention group, 5 patients withdrew; 4 patients withdrew in the control group. Patients in the intervention group had significantly larger improvements in the European Organization for Research and Treatment of Cancer Quality of Life Questionnaire Core 30 total score (mean difference 11.51; 95% CI 10.68-12.35; Cohen *d*=1.73) and Bowel Symptoms Self-Management Behaviors Questionnaire total score (mean difference 8.80; 95% CI 8.28-9.32; Cohen *d*=1.94) than those in the control group. This improvement effect remained stable at 3-month follow-up (mean difference 14.47; 95% CI 13.65-15.30; Cohen *d*=1.58 and mean difference 8.85; 95% CI 8.25-9.42; Cohen *d*=2.23). The LARS score total score had significantly larger decreases after intervention (mean difference –3.28; 95% CI –4.03 to –2.54; Cohen *d*=–0.39) and at 3-month follow-up (mean difference –6.69; 95% CI –7.45 to –5.93; Cohen *d*=–0.69). The Perceived Social Support Scale total score had significantly larger improvements after intervention (mean difference 0.47; 95% CI 0.22-0.71; Cohen *d*=1.81).

**Conclusions:**

Our preliminary findings suggest that the mobile health–based remote interaction management intervention significantly enhanced the self-management behaviors and QoL of patients with LARS, and the effect was sustained. Mobile health–based remote interventions become an effective method to improve health outcomes for many patients with LARS.

**Trial Registration:**

Chinese Clinical Trial Registry ChiCTR2200061317; https://tinyurl.com/tmmvpq3

## Introduction

### Background

The Global Cancer Statistics 2020 showed that colorectal cancer ranks third in incidence of malignant tumors and second in cause of death worldwide [1]. Colorectal cancer incidence is also on the rise in China, with rectal cancer accounting for 60% of cases and middle and lower rectal cancers being the most common [2]. With the advancement of medical technology, optimal management of middle and lower rectal cancers increasingly favors sphincter-preserving surgery (SPS) [3]. This operation preserves anal function and avoids the inconvenience and pressure caused by permanent colostomy [4]. However, 70%-90% of patients after SPS struggle with long-term anorectal functional disturbances called low anterior resection syndrome (LARS) [5,6].

The presence of LARS has a severe adverse effect on the quality of life (QoL) of patients [7]. Postoperative LARS induces a spectrum of adverse physical and psychological effects in patients; for example, up to 50% of patients with LARS report toilet dependence during rehabilitation [8,9], 36% of patients experience pain, and approximately 13% of patients report high psychological distress [10,11]. Furthermore, LARS can restrict a patient’s social life, leading to further impact on their QoL [12]. Recently, longitudinal studies have found that patients’ QoL is still affected by LARS even 15 years after surgery [13]. Research has shown that patients can improve their QoL through methods, such as pelvic floor muscle exercises and dietary adjustments during home care; however, the effectiveness of these methods is limited by patients’ lack of knowledge of LARS and rehabilitation guidance [14,15].

Owing to the frequent occurrence of LARS in patients post discharge, patients must have a high level of self-management behavior [16]. However, in China, the majority of patients have a passive response to LARS, and their self-management behavior is at a low level [17]. Enhancing self-management awareness and providing information on supportive care can improve the self-management behavior of patients with LARS [18]. Research has demonstrated that motivational interviewing (MI) enhances self-management awareness and supports behavioral change [19].

Therefore, to improve patients’ QoL and self-management behaviors, providing supportive care information to patients is crucial. A qualitative exploration of patients with LARS’s perspectives on information needs revealed that timely symptom management measures are critical during home-based rehabilitation [20]. However, it is difficult to maintain continuity and instantaneity with existing management measures [21,22]. Owing to current advances in mobile technology, mobile health (mHealth) has been widely considered a means of patient health management, which can improve the effects of symptoms and assist patients in timely access to the required information [23,24].

To date, remote follow-up tools for patients with LARS have yielded promising results [25]. For patients with LARS, mHealth-based remote interventions may become an effective method to assist them in improving symptoms. However, mHealth intervention measures constructed for patients with LARS are rare. Most studies have only completed the development and pilot research of remote intervention programs, leading to insufficient data on the effectiveness of remote interventions in improving patient health outcomes [26,27]. WeChat (Tencent Corp) is China’s most frequently used instant messaging and social media application [28]. Evidence suggests that WeChat-based mHealth interventions effectively improve health outcomes in various health conditions [29,30].

### Objective

This study aimed to assess the effectiveness of a remote digital management intervention designed for patients with LARS. The effectiveness of the intervention measure is determined by improvement in QoL, self-management behaviors, gastrointestinal symptoms, and social support. We hypothesized that the remote digital management intervention can effectively improve the health outcomes of patients with LARS.

## Methods

### Study Design

This study was conducted from July 15, 2022, to March 15, 2023, in Hefei, China. Our team provided a 3-month intervention and followed up with all patients for an additional 3 months. The intervention group used the “e-bowel safety” applet and received monthly MI. The control group received the usual care and was provided with a handbook containing information related to LARS. The CONSORT (Consolidated Standards of Reporting Trials) checklist is in Multimedia Appendix 1.

### Ethical Considerations

This randomized controlled trial (RCT) was approved by the ethics committee of the First Affiliated Hospital of Anhui Medical University (PJ2022-07-53) and registered on the Chinese Clinical Trial Registry (ChiCTR2200061317). All data were identified with a code number to ensure the confidentiality of the subjects’ data. No compensation was provided to participants.

### Participants

The patients were recruited from a tertiary hospital in Hefei, Anhui Province, China. Patients were eligible to participate in our study if they met the following criteria: age older than 18 years, a diagnosis of rectal cancer, underwent SPS, LARS scores ≥21, ostomy closure surgery performed at least 3 months prior, the ability to read and write text, and proficiency in using WeChat. Patients with chronic gastrointestinal conditions, prior or current mental health disorders, cognitive impairments, communication disorders, or those who have participated in other clinical studies are ineligible for participation in this research. When patients meeting the recruitment criteria appeared in the hospital database, the system sent recruitment information to these patients with the approval of doctors not directly involved in the research design.

In this study, the sample size was determined based on the QoL. Previous research has shown that the QoL for patients with rectal cancer is 77±19 [31]. In an RCT using the EORTC QLQ-C30, a difference of 10 points is considered clinically significant [32]. With a two-sided test level of 0.05 and 80% test efficacy, each group requires a sample size of 45. Accounting for a 20% dropout rate, 112 patients are needed.

### Intervention

Our previous study provided a comprehensive description of the intervention protocol [33]. The patients in the intervention group used the “e-bowel safety” applet for 3 months. They were required to check in on the applet daily and record their daily gastrointestinal symptoms. Our “e-bowel safety” applet comprises 4 main sections: a rehabilitation plan, LARS knowledge, web-based consultation, and patient stories. The rehabilitation plan module involves the collaborative development of home dietary and exercise plans by patients and researchers. The applet features intelligent reminders to monitor daily plan completion and provide prompts. After completing the rehabilitation plan, patients must fill out a daily health diary, and researchers dynamically adjust the rehabilitation plan based on patients’ feedback and physical condition. The LARS knowledge module offers evidence-based information on LARS and symptom management strategies. The web-based consultation module provides patients with an opportunity to interact with health care professionals, offering personalized guidance and feedback. The patient stories module allows patients to share symptom management experiences or engage with other patients, with all published content subject to researcher approval. Additionally, an incentive system has been designed to encourage participation. For instance, patients earn points by sharing personal stories or comments, which can later be exchanged for rewards after accumulating a certain number of points.

Moreover, our team members conducted monthly MIs with patients. MIs were led by 4 researchers with expertise in health coaching and disease management, including 1 clinical psychologist (Shangxin Zhang) and 3 registered nurses (TW, HH, and Ling Fang). The researchers engage with patients via WeChat for 30-60 minutes per call. The aim of MIs is to assist patients in setting rehabilitation goals, reinforcing self-management awareness, and promoting health behavior changes. The content of MIs is based on the interview guide determined by the research team, which guides the conversation from the initial session to explore the participant’s motivation to identify the facilitating factors and barriers to achieving their health goals. The interview guide is outlined in Multimedia Appendix 2.

Patients in the control group received the usual care and were provided with a handbook containing information related to LARS. At the same time, our team members followed up with patients, using the same timing and frequency as the MI intervention group.

### Randomization and Masking

This study was a single-blind, two-arm RCT. After obtaining consent from eligible patients, assistants who were not involved in the study randomly assigned them to the intervention and control groups at a 1:1 ratio. The randomization process was performed by the assistants and anonymized envelopes were used with block randomization, including block sizes randomly varying between 4 (2:2) and 6 (3:3). The research assistants (Ping Ni and Ai Wang) who collected the data were unaware of the patient assignments throughout the study. Patients used the QR codes provided by the research team to access the “e-bowel safety” applet, effectively reducing contamination between the 2 groups. Patients were blinded to their group assignments throughout the entire research process.

### Quality Control and Participant Retention

Several strategies were used to ensure quality control and participant retention. Our “e-bowel safety” applet can monitor patients’ plan execution and provide reminders, which ensures the daily plans are followed strictly by patients. Before the formal intervention, we conducted a pilot experiment and gathered participant feedback to enhance our plan. The specific results are included in Multimedia Appendix 3. Furthermore, patients received consistent guidance from our research assistants (Ping Ni and Ai Wang) when they had questions about the questionnaire content. Before the start of the study, all research assistants must undergo training and assessment on the use of all questionnaires by research team members. Only research assistants who pass the assessment can participate in data collection. Additionally, team members regularly check the progress of research assistants’ work to ensure that they are following the questionnaire collection process, identifying issues promptly, and making corrections.

### Outcome Measures

The patients’ demographic and clinical information were obtained from the hospital database. Data were collected from patients using scales for their QoL, social support, self-management behaviors, and LARS scores at different time periods (0, 3, and 6 months). The research assistants (Ping Ni and Ai Wang) who collected the data assisted patients in completing questionnaires over the phone or through direct personal interaction.

### Primary Outcome: QoL

The EORTC-QLQ-C30 (European Organization for Research and Treatment of Cancer Quality of Life Questionnaire Core 30) was used to measure QoL. This questionnaire comprises 30 items divided into 15 dimensions, including 1 dimension for QoL, 5 dimensions for functionality, 3 dimensions for symptoms, and 6 dimensions for additional symptoms. All dimension scores were linearly transformed to a scale of 0-100 points. Elevated scores on the 5 functionality dimensions and the QoL dimension were linked to improved functional status, whereas the reverse pattern was observed for the symptom dimensions and additional symptom dimensions. The Cronbach α coefficient ranged from 0.764 to 0.809 [34].

### Secondary Outcome

#### Self-Management

The self-management behavior of patients was assessed by the Bowel Symptoms Self-Management Behaviors Questionnaire (BSSBQ). This questionnaire comprises 24 items divided into 5 functional scales, with each item scored on a scale of 0 (never) to 7 (always). Higher scores indicate better bowel symptom self-management behavior. The Cronbach α coefficient was 0.81 [17].

#### Bowel Function

The LARS score consists of 5 items, with a total score ranging from 0 to 42. Patients’ gastrointestinal symptoms are classified into no LARS, minor LARS, and major LARS based on the total score. The LARS score is a validated instrument for assessing bowel symptoms. The Cronbach α coefficient was 0.767 [35].

#### Social Support

The Perceived Social Support Scale (PSSS) consists of 12 items, with each item scored on a scale of 1 (extreme disagreement) to 7 (strong consent). The total scores ranged from 12 to 84. The higher the score, the stronger the perceived social support by the patient. This scale is widely used to assess the level of social support among patients in China. The Cronbach α coefficient of this scale was 0.899 [36].

### Feasibility

The feasibility of intervention was assessed through the completion status of MI sessions and the adherence to health diary entries. The 3-month intervention corresponds to 3 MI sessions and 84 days of health diary entries.

### Statistical Methods

All data were analyzed using SPSS Statistics (version 23.0; IBM Corp). An intention-to-treat analysis was performed in this study. We used the last observed values of the patients to replace missing data. Chi-square analysis was used to analyze the remaining demographic characteristics, and a 2-tailed independent sample *t* test was used to analyze the age and tumor height. Descriptive data were computed, including means with SD, medians with ranges, and frequencies with proportions where appropriate. The statistical significance was established at *P*<.05 (2-tailed test). Generalized estimating equations were used to analyze changes in QoL, self-management behaviors, LARS, and social support scores at different time points. The calculation of effect sizes was performed using Cohen *d* for the mean differences at various time periods.

## Results

### Participant Characteristics

**Figure 1:**
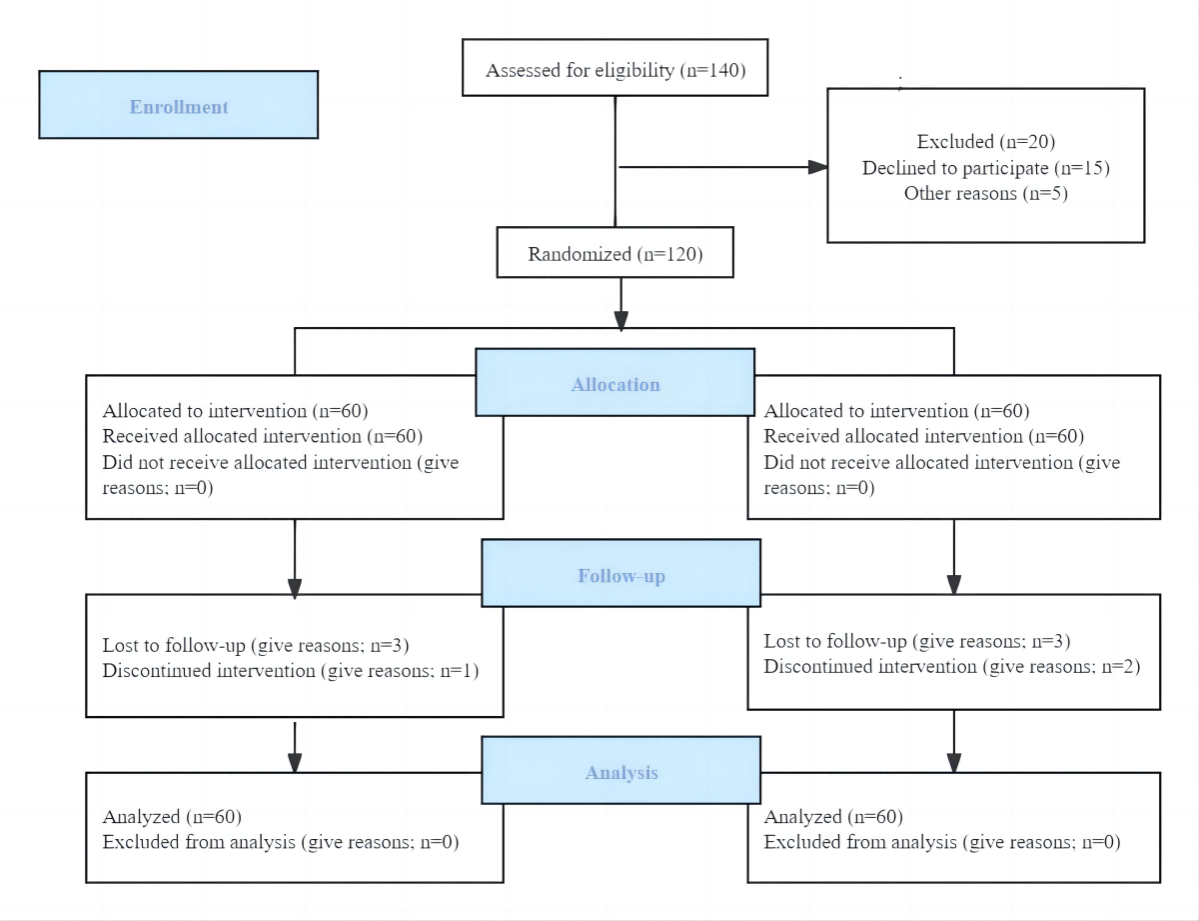
CONSORT (Consolidated Standards of Reporting Trials) flowchart.

Initially, 60 patients were recruited in the control and intervention groups. During the study, 9 patients dropped out (dropout rate 7.5%). In the intervention group, 5 patients withdrew from the study, including 2 patients who received a reostomy because of an anastomotic fistula and 3 patients whose condition worsened. In the control group, 4 patients dropped out, including 2 patients whose condition worsened and 2 patients who refused to continue the intervention because of the side effects of chemotherapy. No statistically significant differences were observed between the patients who dropped out and those who completed all evaluations (*P*=.17). Figure 1 shows the CONSORT flowchart of this study. Table 1 demonstrates no statistically significant differences in the demographics and clinical information between the control and intervention groups at baseline.

**Table 1:** Basic participant characteristics of the 2 groups.

Characteristics			Intervention group (n=60)		Control group (n=60)		*t* test (*df*) or chi-square value (*df*)			*P* value
**Sex, n (%)**								0.93 (1)		.34
	Male	42 (70)		37 (62)						
	Female	18 (30)		23 (38)						
Age (years), mean (SD)			62.72 (7.91)		61.78 (11.80)		0.51 (118)			.61
**Education, n (%)**								0.07 (2)		.96
	Junior high school or lower	33 (55)		32 (53)						
	High school	19 (32)		19 (32)						
	College or higher	8 (13)		9 (15)						
**Marital status, n (%)**								0.21 (1)		.65
	Married	58 (97)		57 (95)						
	Single	2 (3)		3 (5)						
**Tumor stage, n (%)**								1.42 (3)		.70
	I	14 (23)		13 (22)						
	II	24 (40)		30 (50)						
	III	20 (33)		15 (25)						
	IV	2 (4)		2 (3)						
Tumor height, mean (SD)			7.62 (1.708)		7.80 (1.811)		–0.57 (118)			.57
**Postoperative time (months), n (%)**								0.378 (2)		.83
	<6	18 (30)		17 (28)						
	6-12	27 (45)		25 (42)						
	>12	15 (25)		18 (30)						
**Surgical procedures, n (%)**								0.24 (1)		.62
	Laparoscopy	51 (85)		49 (82)						
	Laparotomy	9 (15)		11 (18)						
**Surgical approach, n (%)**								0.34 (1)		.56
	LAR^a^	58 (9)		59 (98)						
	TaTME^b^	2 (3)		1 (2)						
**Temporary stoma, n (%)**								0.53 (1)		.47
	Yes	29 (48)		33 (55)						
	No	31 (52)		27 (45)						
**Chemotherapy, n (%)**								0.88 (2)		.65
	Preoperative	8 (13)		5 (8)						
	Postoperative	49 (82)		51 (85)						
	No	3 (5)		4 (7)						
**Residence, n (%)**								1.20 (1)		.27
	Countryside	28 (47)		34 (57)						
	City	32 (53)		26 (43)						
**Measurements, mean (SD)**										
	EORTC-QLQ-C30^c^	69.67 (4.26)		69.42 (3.66)		0.35 (118)			.72	
	BSSBQ^d^	30.33 (1.90)		30.58 (2.01)		–0.70 (118)			.49	
	LARS^e^ score	31.07 (3.88)		31.32 (4.73)		–0.32 (118)			.75	
	PSSS^f^	34.42 (1.62)		34.3 (1.48)		0.29 (118)			.77	

^a^LAR: low anterior resection.

^b^TaTME: transanal total mesorectal excision.

^c^EORTC-QLQ-C30: European Organization for Research and Treatment of Cancer Quality of Life Questionnaire Core 30.

^d^BSSBQ: Bowel Symptoms Self-Management Behaviors Questionnaire.

^e^LARS: Low anterior resection syndrome.

^f^PSSS: Perceived Social Support Scale.

### Main Evaluation Indexes

Table 2 shows that the patients’ QoL improved for both groups. Patients in the intervention group demonstrated greater improvements in the EORTC-QLQ-C30 total score than those in the control group after intervention (mean difference 11.51; 95% CI 10.68-12.35; Cohen *d*=1.73). Furthermore, this improvement effect remained stable at 3-month follow-up (mean difference 14.47; 95% CI 13.65-15.30; Cohen *d*=1.58). Table 3 shows that the EORTC-QLQ-C30 total score in both groups exhibited a trend of change over the 6-month period (*P*<.001). Differences were observed between the 2 groups and the interaction between group and time. A subgroup analysis was conducted on patients receiving preoperative chemotherapy versus postoperative chemotherapy. Among the 49 patients in the intervention group and 51 in the control group undergoing postoperative chemotherapy, a nominally significant improvement in the change from baseline in the EORTC-QLQ-C30 total score at 3 months was observed compared to the control group (difference of 4.42; *P*<.001). However, this effect was not seen in patients receiving preoperative chemotherapy. The specific results are included in Multimedia Appendix 4.

**Table 2:** Comparison of the outcomes between the 2 groups after the intervention and at 3-month follow-up.

Outcomes		Intervention group, mean (SD)	Control group, mean (SD)	Cohen *d*	GEE^a^ statistical tests	
					Score, (95% CI)^b^	*P* value
**EORTC-QLQ-C30^c^**						
	T0^d^	69.67 (4.26)	69.42 (3.66)	N/A^e^	N/A	N/A
	TI^f^	83.41 (2.46)	78.71 (2.72)	1.73	11.51 (10.68 to 12.35)	<.001
	T2^g^	86.22 (2.49)	81.82 (2.79)	1.58	14.47 (13.65 to 15.30)	<.001
**BSSBQ^h^**						
	T0	30.33 (1.90)	30.58 (2.01)	N/A	N/A	N/A
	TI	41.23 (2.26)	37.28 (2.04)	1.94	8.80 (8.28 to 9.32)	<.001
	T2	42.25 (2.58)	36.37 (2.63)	2.23	8.85 (8.25 to 9.42)	<.001
**LARS^i^ score**						
	T0	31.07 (3.88)	31.32 (4.73)	N/A	N/A	N/A
	TI	26.95 (3.51)	28.87 (4.83)	–0.39	–3.28 (–4.03 to 2.54)	<.001
	T2	22.87 (3.09)	26.13 (4.67)	–0.69	–6.69 (–7.45 to 5.93)	<.001
**PSSS^j^**						
	T0	34.42 (1.62)	34.3 (1.48)	N/A	N/A	N/A
	TI	36.63 (1.44)	33.05 (1.98)	1.81	0.47 (0.22 to 0.71)	<.001
	T2	34.80 (1.19)	34.40 (1.55)	0.25	0.23 (–0.20 to 0.45)	.07

^a^GEE: Generalized estimating equations.

^b^Difference in mean change from baseline to endpoint between the groups.

^c^EORTC-QLQ-C30: European Organization for Research and Treatment of Cancer Quality of Life Questionnaire Core 30.

^d^Baseline.

^e^N/A: Not applicable.

^f^After the intervention.

^g^3-month follow-up.

^h^BSSBQ: Bowel Symptoms Self-Management Behavior Questionnaire.

^i^LARS: Low anterior resection syndrome score.

^j^PSSS: Perceived Social Support Scale.

**Table 3:** Variation tendency of the EORTC-QLQ-C30^a^, BSSBQ^b^, LARS^c^ score, and PSSS^d^ in the 2 groups.

Outcomes	Group effect		Time effect		Group×time	
	*F* test (*df*)	*P* value	*F* test (*df*)	*P* value	*F* test (*df*)	*P* value
EORTC-QLQ-C30	68.50 (1)	<.001	53.81 (2)	<.001	27.79 (2)	<.001
BSSBQ	48.15 (1)	<.001	74.31 (2)	<.001	3.24 (2)	.03
LARS Score	7.78 (1)	.05	74.94 (2)	<.001	21.34 (2)	<.001
PSSS	29.97 (1)	<.001	14.47 (2)	.001	71.71 (2)	<.001

^a^EORTC-QLQ-C30: European Organization for Research and Treatment of Cancer Quality of Life Questionnaire Core 30.

^b^BSSBQ: Bowel Symptoms Self-Management Behaviors Questionnaire.

^c^LARS: Low anterior resection syndrome.

^d^PSSS: Perceived Social Support Scale.

### Secondary Evaluation Indexes

Table 2 shows that the patients’ self-management behavior was enhanced for both groups. The BSSBQ total score had significantly larger improvements after intervention (mean difference 8.80; 95% CI 8.28-9.32; Cohen *d*=1.94) and at 3-month follow-up (mean difference 8.85; 95% CI 8.25-9.42; Cohen *d*=2.23) between groups. The BSSBQ total score showed statistically significant time effects (*P*<.001; Table 3).

The LARS score total score had significantly larger decreases after intervention (mean difference –3.28; 95% CI –4.03 to –2.54; Cohen *d*=–0.39) and at 3-month follow-up (mean difference –6.69; 95% CI –7.45 to –5.93; Cohen *d*=–0.69). Table 3 shows that the LARS score total score in both groups exhibited a trend of change over the 6-month period. The intergroup effect exhibits homogeneity (*P*=.05).

The PSSS total score had significantly larger improvements after intervention (mean difference 0.47; 95% CI 0.22-0.71; Cohen *d*=1.81); however, the improvement in this effect did not persist at 3-month follow-up (mean difference 0.23; 95% CI –0.20 to 0.45; *P*=.07; Table 2). Table 3 shows that the PSSS total score in both groups exhibited a trend of change over the 6-month period.

### Feasibility

Among the 55 patients who completed the intervention, 45 patients completed 3 MI sessions on time, 7 patients postponed 1 MI session because of scheduling conflicts, and 3 patients only completed 2 MI sessions. The mean number of attended MI sessions was 2.95 (SD 0.23). Additionally, 40 patients completed 84 health diary entries, while the remaining 11 patients did not submit completed entries or fulfill the required entries. The mean number of days of health diary entries was 82.87 (SD 3.15). We invited patients from the intervention group to complete a survey to evaluate their perceptions of the intervention's usability. In the end, 49 people completed the survey. The specific results are included in Appendix 5.

## Discussion

### Principal Findings

To the best of our knowledge, the “e-bowel safety” applet is the first mobile app developed for patients with LARS in China. This study offers a valuable reference point for future initiatives in mHealth interventions for patients with LARS. A mHealth-based intervention was found to be feasible and effective in helping patients with LARS relieve bowel dysfunction, improve their self-management behavior, and improve their QoL compared to usual care.

This study found that the EORTC-QLQ-C30 total score of the intervention group increased significantly more than that of the control group after the intervention, indicating that the mHealth-based remote interaction could improve the QoL of patients with LARS. These results can be attributed to multiple factors. First, uncontrollable changes in intestinal function, concerns about prognosis, and fear of the future make patients with LARS feel uncertain [37]. A sense of uncertainty influences a patient’s QoL [38]. Patients using the “e-bowel safety” applet can provide timely feedback on their problems to the medical staff and obtain solutions, which can effectively reduce the uncertainty of patients during home rehabilitation. Second, decreased bowel dysfunction severity positively affected the QoL [39]. Third, peer support reportedly enhances cancer adaptation and QoL [40]. The patients’ stories module offers a channel for communication and emotional support among patients with LARS. In this section, patients can share their experiences related to disease management or self-management and receive responses from their peers through comments.

As expected, the BSSBQ total score in the intervention group after the intervention was significantly higher than that in the control group. The findings supported our hypothesis that health-based remote interaction can enhance the self-management behavior of patients with LARS. After the intervention, the results of enhanced self-management behavior were consistent with a previous face-to-face 6-month self-management program study for LARS, which may indicate that mHealth-based remote interaction may yield intervention effects on self-management behavior similar to those observed in face-to-face interventions [41]. However, a more significant effect was observed at 3-month follow-up. This may be because monthly motivational interviews help patients adopt positive health behaviors and improve their self-management awareness [42]. Moreover, current web-based self-management information on LARS is overly intricate for patients, and the information fails to meet the patient’s needs [43]. The strength of our “e-bowel safety” applet is the credibility of the information provided and medical consultation from experts, which can meet the information needs of patients. Finally, our team members created an individualized self-management plan for each participant in the intervention group and reminded them to follow the plans on the applet, which ensured that the patients developed good habits.

Consistent with previous studies [41], this study found that the intervention group demonstrated a more significant decline in the LARS score than the control group. The LARS score also showed significant time effects, indicating that the patient’s bowel dysfunction changed significantly during the 6-month period. This may be because our team members guided patients in rehabilitation exercises and diet adjustments, which have been proven effective in improving bowel dysfunction [44-46]. Meanwhile, the severity of bowel dysfunction decreased over time [13].

Unlike those of previous studies, our findings indicated that mHealth-based remote interaction management intervention could improve the social support levels in the short term; however, sustaining a stable long-term effect on social support was not realized [47]. The patients in the study might have used the “e-bowel safety” applet only for 3 months, and the impact of the intervention on social support may not yield a residual advantage at 3-month follow-up. Furthermore, most patients’ physical and social functions gradually stabilized at 6 months. Our “e-bowel safety” applet focuses on intensive support for symptom management and lacks support knowledge for patients when symptoms plateau, which should be refined in future studies to achieve long-term effects.

In this study, MI was used to stimulate behavioral change and maintenance. The dual intervention of mHealth and MI promotes effective engagement and motivation for health behavior changes. Nearly all the patients (55/60) successfully completed the 3-month intervention and the follow-up during the intervention process, signifying that the mHealth-based remote interaction management intervention is feasible and acceptable. In addition, none of the patients in the intervention group experienced adverse consequences caused by the intervention, indicating that the intervention was safe.

### Limitations

This study has some limitations. First, this study enrolled patients from a tertiary hospital in China, which restricts the generalizability of our results. In the future, we will recruit patients from more hospitals to confirm our research findings. Second, patients were subjected to a limited 3-month follow-up period, thereby restricting our assessment of the enduring effects of the mHealth-based remote interaction management intervention on self-management behavior and QoL. Finally, patients were required to use WeChat and smartphones, which presents the potential for selection bias.

### Conclusions

The mHealth-based remote interaction management intervention effectively enhanced the self-management behavior and QoL of patients with LARS, and the impact remained consistent during the 3-month follow-up. Bowel dysfunction also significantly improved throughout the entire research process. This study suggests that mHealth intervention could provide an effective and new option for many patients with LARS. Multicenter studies are necessary to establish the generalizability and effectiveness of these interventions.

## Appendix

Multimedia Appendix 1 CONSORT-EHEALTH (Consolidated Standards of Reporting Trials of Electronic and Mobile HEalth Applications and onLine TeleHealth) checklist (version 1.6.1).

Multimedia Appendix 2 The interview guide of motivational interviewing.

Multimedia Appendix 3 Results of pilot experiment.

Multimedia Appendix 4 The results of subgroup analysis.

Multimedia Appendix 5 Comments and attitudes towards intervention of intervention group.

## Abbreviations

BSSBQ: Bowel Symptoms Self-Management Behaviors Questionnaire
CONSORT: Consolidated Standards of Reporting Trials
EORTC-QLQ-C30: European Organization for Research and Treatment of Cancer Quality of Life Questionnaire Core 30
LARS: low anterior resection syndrome
mHealth: mobile health
MI: motivational interviewing
PSSS: Perceived Social Support Scale
QoL: quality of life
RCT: randomized controlled trial
SPS: sphincter-preserving surgery
