# Supplementary material for: Effect of a Mobile Health–Based Remote Interaction Management Intervention on the Quality of Life and Self-Management Behavior of Patients With Low Anterior Resection Syndrome: Randomized Controlled Trial
Source: J Med Internet Res. 2024 Aug 13;26:e53909. doi: 10.2196/53909 (PMC11350307; doi:10.2196/53909)
Supplement: Multimedia Appendix 2 [file jmir_v26i1e53909_app2.docx]

**Section S1: Self-Reflection and Motivation**

1.Are you currently experiencing any gastrointestinal symptoms? Which self-management knowledge do you believe has helped improve your symptoms? What specific self-management knowledge do you think is essential for controlling your gastrointestinal symptoms?

2.How has practicing self-management impacted you?

3.How do you plan to incorporate self-management into your daily life? Will you create detailed plans for this?

4.What is the most important self-management behavior for you right now?

5.How do these perspectives shape your future and influence your lifestyle choices?

**Section S2: Bridging the Gap and Looking to the Future**

1.How do you think your current gastrointestinal symptoms will affect your life after you are discharged?

2.What do you think the worst outcome could be if you don't follow the medical staff's instructions on managing gastrointestinal symptoms at home?

3.Conversely, what do you think the best result could be if you make lifestyle changes according to the instructions?

4.How much control do you hope to have over your gastrointestinal symptoms?

**Section S3: Prompting Decision-Making and Eliciting Hope**

1.Have you made plans for managing your gastrointestinal symptoms, such as controlling your diet and engaging in physical activities?

2.How do you plan to ensure you stick to your self-management plan?

3.Please describe in detail how you plan to carry out these behaviors, including when you will do them.

4.What factors do you think might prevent you from completing your self-management plan on time?

5.What strengths do you have that will help you succeed? For example, past lifestyle habits and communication skills.

**Section S4: Strengthening Commitment and Supporting Change**

1.What situations in the past month might hinder your ongoing self-management of gastrointestinal symptoms?

2.Do you believe you can overcome these obstacles? What steps will you take?

3.What achievements have you doubted in the past but eventually accomplished? 4.How did you stay committed? Do you trust in using the same approach for self-management?

5.What goals do you plan to set through self-management behavior?
