# Supplementary material for: Effect of a Mobile Health–Based Remote Interaction Management Intervention on the Quality of Life and Self-Management Behavior of Patients With Low Anterior Resection Syndrome: Randomized Controlled Trial
Source: J Med Internet Res. 2024 Aug 13;26:e53909. doi: 10.2196/53909 (PMC11350307; doi:10.2196/53909)
Supplement: Multimedia Appendix 3 [file jmir_v26i1e53909_app3.docx]

We invited 20 patients with LARS to participate in our pilot study, with 1 person dropping out due to worsening condition and 2 refusing to continue the intervention, leaving 17 people who completed all interventions. We conducted interviews with all patients except for one who dropped out due to worsening condition.

Basic participant characteristics.

| Characteristics | N=20 |
| --- | --- |
| Sex, n(%) |  |
| Male | 13 (65.0) |
| Female | 7 (35.0) |
| Age, mean (SD) | 61.69 (11.12) |
| Education, n(%) |  |
| Junior high school or lower | 11 (55.0) |
| High school | 6 (30.0) |
| College or higher | 3 (15.0) |
| Marital status,n(%) |  |
| Married | 19 (95.0) |
| Single | 1 (5.0) |
| Tumor stage, n(%) |  |
| I | 7 (35.0) |
| II | 10 (50.0) |
| III | 3 (15.0) |
| IV | 0 (0) |
| Tumor height, mean (SD) | 7.73 (1.88) |
| Postoperative time |  |
| <6 months | 5 (25.0) |
| 6-12 months | 7 (35.0) |
| >12 months | 8 (40.0) |
| Surgical procedures, n(%) |  |
| Laparoscopy | 18 (90.0) |
| Laparotomy | 2 (10.0) |
| Surgical approach, n(%) |  |
| LAR | 17 (85.0) |
| TaTME | 3 (15.0) |
| Temporary stoma, n(%) |  |
| Yes | 5 (25.0) |
| No | 15 (75.0) |
| Chemotherapy,n(%) |  |
| Preoperative | 3 (15.0) |
| Postoperative | 16 (80.0) |
| No | 1 (5.0) |
| Residence,n(%) |  |
| Countryside | 7 (35.0) |
| City | 13 (65.0) |

**Experience of the intervention**

All participants expressed that the intervention helped them address issues encountered in their daily lives. They believed that participating in the intervention could alleviate psychological stress and motivate them to follow their daily training plans.

"After coming home, I no longer feel lost about what to do every day. Sometimes, reading the experiences shared by others on the platform makes me find it quite interesting." "At home, I sometimes feel quite helpless. Your reminders about diet and exercise through this app often warm my heart." "At home, I used to get scared when needing to pass stool frequently, thinking it might be a recurrence. But after reading the information on the platform, I felt less anxious."

Multiple participants mentioned that participating in the intervention helped supervise their daily home exercises.

"I tend to lose motivation for doing pelvic floor exercises if I do them for too long. Fortunately, your reminders keep me on track." "If I miss a workout, the platform prompts me to stay committed to my daily goals."

Several participants found the intervention content rich and easy to understand.

"My eyesight isn't as good as before due to my age, but the option to have the content read aloud is very convenient." "When I was discharged, I was taught how to eat and exercise at home. It feels like all the aspects needing attention were considered." "I just wanted to know how to eat well at home, and the platform's food recommendations with images are quite helpful."

Additionally, the intervention strengthened communication with healthcare providers

Through this, I can easily reach out to you in case of any problems, sometimes avoiding unnecessary hospital visits." "I can ask anything I want to know, it feels very convenient."

**Suggestions for the intervention**

The participants believe that the time for MI sessions can be adjusted.

"Typically, weekday mornings are the busiest, so afternoons would be more convenient." "Conducting MI sessions on non-working days would provide more time for communication."

Participants also emphasize the importance of necessary motivation.

"Can a points system be added? This way, I would feel more motivated to share stories or interact with other patients at times." "If there were points for filling out the health diary each time, I would be more willing to do so."

Participants recommend adding an offline mode to the app so that knowledge resources can be downloaded for offline browsing, enabling access without an internet connection.

"Sometimes the internet connection is poor, it would be great to have an offline mode."
