# Supplementary material for: Effect of a Mobile Health–Based Remote Interaction Management Intervention on the Quality of Life and Self-Management Behavior of Patients With Low Anterior Resection Syndrome: Randomized Controlled Trial
Source: J Med Internet Res. 2024 Aug 13;26:e53909. doi: 10.2196/53909 (PMC11350307; doi:10.2196/53909)
Supplement: Multimedia Appendix 4 [file jmir_v26i1e53909_app4.docx]

Subgroup Analysis

| Variable  Subgroup  Group | N | Change from Baseline Mean (SE) | Score (95% Cl) | *P* value |
| --- | --- | --- | --- | --- |
| Chemotherapy |  |  |  |  |
| Preoperative |  |  | 0.58 (-5.08–6.24) | 0.826 |
| Intervention group | 8 | 14.55 (5.48) |  |  |
| Control group | 5 | 13.97 (1.83) |  |  |
|  |  |  |  |  |
| Postoperative |  |  | 4.42 (2.79–6.04) | <.001 |
| Intervention group | 49 | 13.04 (4.54) |  |  |
| Control group | 51 | 8.63 (3.61) |  |  |
