# Supplementary material for: Effect of a Mobile Health–Based Remote Interaction Management Intervention on the Quality of Life and Self-Management Behavior of Patients With Low Anterior Resection Syndrome: Randomized Controlled Trial
Source: J Med Internet Res. 2024 Aug 13;26:e53909. doi: 10.2196/53909 (PMC11350307; doi:10.2196/53909)
Supplement: Multimedia Appendix 5 [file jmir_v26i1e53909_app5.docx]

| Variables | N (%) |
| --- | --- |
| Convenience |  |
| Very inconvenient | 1(2.1) |
| Not very convenient | 2(4.1) |
| Fairly convenient | 5(10.2) |
| Relatively convenient | 24(48.9) |
| Very convenient | 17(34.7) |
| Credibility |  |
| Not credible | 2(4.1) |
| Not very credible | 2(4.1) |
| Fairly credible | 8(16.3) |
| Relatively credible | 16(32.6) |
| Very credible | 21(42.9) |
| Willingness to recommend to those in need |  |
| Not recommended | 1(2.1) |
| Not very recommended | 1(2.1) |
| Fairly recommended | 10(20.3) |
| Relatively recommended | 15(30.6) |
| Highly recommended | 22(44.9) |
| Necessity for widespread promotion |  |
| Very unnecessary | 0(0) |
| Not very necessary | 3(6.1) |
| Fairly necessary | 10(20.3) |
| Relatively necessary | 17(34.7) |
| Very necessary | 19(38.9) |
| Satisfaction |  |
| Very dissatisfied | 1(2.1) |
| Not very satisfied | 2(4..1) |
| Fairly satisfied | 10(20.3) |
| Relatively satisfied | 14(28.6) |
| Very satisfied | 22(44.9) |
